# A Rare Case of First-Time Seizure Induced by Cerebral Venous Sinus Thrombosis Following the Use of Tranexamic Acid for Menorrhagia

**DOI:** 10.3390/reports8040210

**Published:** 2025-10-20

**Authors:** Jennifer Bandt, Emmanuel O. Oisakede, Natalie Walker

**Affiliations:** 1 General/Acute Medical Unit, Bolton NHS Foundation Trust, Bolton BL4 0JR, UK; natalie.walker@boltonft.nhs.uk; 2 Department of Clinical Oncology, Leeds Teaching Hospitals Trust, Leeds LS1 3EX, UK; emmanuel.oisakede@gmail.com; 3 Department of Health Research, University of Leeds, Leeds LS2 9JT, UK

**Keywords:** tranexamic acid, cerebral venous thrombosis, seizures, menorrhagia, cerebrovascular imaging

## Abstract

**Background and clinical significance:** Tranexamic acid (TXA) is commonly used for menorrhagia. Common side effects include diarrhoea, nausea, and vomiting. However, more serious and rare side effects, including embolism, thrombosis, and seizures, are less commonly considered. **Case presentation:** We report the case of a 39-year-old woman of Asian origin who presented after a first-time seizure while driving, following starting tranexamic acid for menorrhagia seven days prior. She complained of a headache, nausea, neck stiffness, floaters, and blurred vision. Her lactate was elevated on presentation. On examination there were no neurologic abnormalities. A computed tomography (CT) head scan showed acute haemorrhagic foci along the left temporal lobe. This prompted a CT venography, which showed filling defects in the left transverse and sigmoid sinuses, in keeping with cerebral venous sinus thrombosis. MRI of the head further showed a blooming artefact, indicating secondary thrombosis of the lateral tentorial sinus on the left side extending into the vein of Labbe. Following the diagnosis of cerebral venous sinus thrombosis, the patient was started on regular levetiracetam as well as a therapeutic dose of low molecular weight heparin. Since the initial episode, she has been seizure-free for over three months now. **Conclusions:** This case highlights the importance of considering less common side effects of tranexamic acid in patients who are taking TXA and are presenting with first-time seizures and headaches. These patients should be monitored for embolic-related intracranial events. A careful diagnostic approach, including cerebrovascular imaging, is essential for an accurate diagnosis and effective treatment.

## 1. Introduction and Clinical Significance

Tranexamic acid (TXA) is a synthetic antifibrinolytic agent widely used in the management of menorrhagia, particularly in cases associated with uterine fibroids [1]. TXA has the ability to stabilise clots by inhibiting the activation of plasminogen to plasmin, thus reducing menstrual blood loss [2]. Beyond gynaecological applications, TXA has established utility in a range of clinical scenarios. These include the management of hereditary angioedema, epistaxis, and the prevention and treatment of significant haemorrhage following major trauma or head injury and postpartum haemorrhage [2,3,4].

While TXA is generally well tolerated, common side effects include mild gastrointestinal symptoms such as nausea, vomiting, and diarrhoea [2]. However, concerns regarding rare but serious adverse effects have been raised, notably the risk of thromboembolic events and seizures. A comprehensive systematic review by Murao et al. [5] found no significant increase in thrombotic events among bleeding patients treated with TXA. However, it noted a dose-dependent increase in the risk of seizures, particularly in patients receiving high-dose intravenous TXA, a finding that has been attributed to TXA’s inhibitory action on γ-aminobutyric acid (GABA) and glycine receptors in the central nervous system [6,7]. This conclusion aligns with another large-scale systematic review by Taeuber et al. [8]. Their analysis further reinforced the safety of TXA with respect to thrombotic risk, especially when used at standard therapeutic doses in the context of acute bleeding. It is safe to say TXA has no independent risk of thrombotic events.

Nevertheless, isolated case reports and pharmacovigilance data have documented associations between TXA and thrombotic complications, including myocardial infarction, pulmonary embolism, deep vein thrombosis, and thrombotic stroke [9,10,11,12]. Notably, reports of TXA-induced cerebral venous sinus thrombosis are exceedingly rare, with only a few published cases to date, indicating a need for ongoing surveillance and case reporting to better understand this potential risk.

## 2. Case Presentation

The patient is a 39-year-old woman of Asian origin who was brought into the emergency unit by an ambulance after seizure-like activity while driving. She had no significant past medical history and was not on any chronic medications. It is worth mentioning that she was initially on norethisterone, a progesterone-only contraceptive pill, but this was discontinued by the patient five weeks prior to presentation as it did not improve her menorrhagia.

She was started on TXA by her general practitioner (GP) for fibroids and menorrhagia one week prior to presentation. As the patient did not experience improvement after completing an initial four-day course of TXA, she was prescribed another three-day course. She took 1 g three times a day for the period she was on it. Since starting the second course, she had developed the ‘worst headache of her life’. The headache was described as frontal and left-sided throbbing. It reached maximum intensity with a strength of 10/10 within minutes and radiated down her neck. There was associated nausea, and the patient complained of blurred vision, floaters, and neck stiffness. There was no relief from simple analgesia, but eventually the pain settled, although it did not disappear completely.

On the day of the presentation (the third day from the onset of headache), the patient was driving when she developed a seizure. This was witnessed by a passenger in the same vehicle, who described it as a generalised tonic–clonic seizure. This led to a low-impact crash into a stationary vehicle in front. No injury was sustained, and no airbag was deployed. Following the event, no neurological sequelae were identified other than post-ictal confusion, which settled upon arrival at the emergency unit. See Table 1 for a chronological overview of symptom evolution until emergency presentation.

Upon presentation, her blood results revealed mildly elevated inflammatory markers, iron deficiency anaemia, and lactatemia. Detailed blood results at presentation are shown in Table 2. Further coagulation screening, including tests for Factor V Leiden mutation, prothrombin mutation, antithrombin III deficiency, protein C and S, JAK-2 mutation, and anti-beta-2 glycoprotein 1 antibodies (both IgM and IgG), were all negative.

**Table 2:** Blood test results at presentation.

Parameter	Result	Reference Range
Haemoglobin (Hb)	77 g/L	115–165 g/L
Mean Corpuscular Volume (MCV)	69.9 fL	79–100 fL
Ferritin	11 µg/L	13–150 µg/L
Serum Iron	2 µmol/L	5.8–34.4 µmol/L
Transferrin Saturation	3%	20–55%
Total Iron Binding Capacity (TIBC)	72 µmol/L	45–72 µmol/L
White Cell Count (WCC)	16.5 × 10^9^/L	4.0–11.0 × 10^9^/L
Neutrophils	13.9 × 10^9^/L	1.7–7.5 × 10^9^/L
Platelets	363 × 10^9^/L	150–400 × 10^9^/L
C-Reactive Protein (CRP)	16.3 mg/L	<5 mg/L
Lactate (Blood Gas)	4.9 mmol/L	0.5–2.2 mmol/L

**Figure 1:**
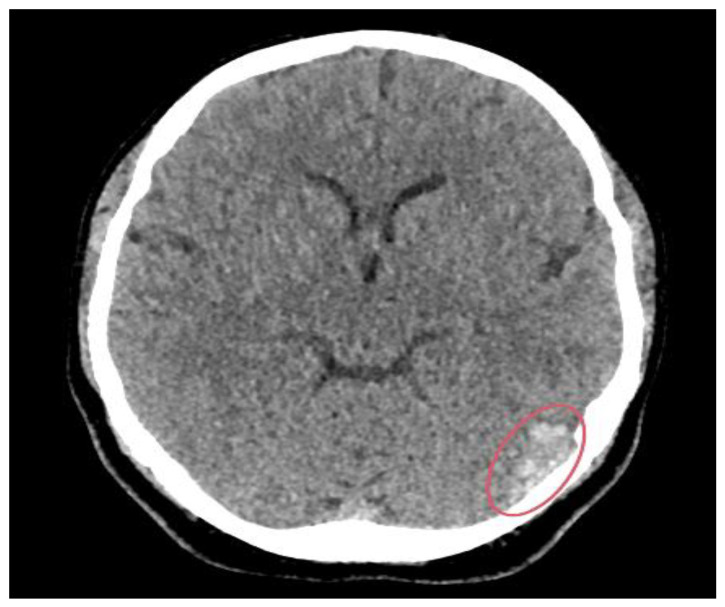
CT brain scan showing hyperdensities in the left temporal lobe (red circle), indicating acute haemorrhagic foci.

The patient had a CT head scan, which revealed acute haemorrhagic foci along the left temporal lobe (Figure 1).

The neurosurgical and neurological team was consulted, and they advised starting the patient on levetiracetam 500 mg twice daily and doing a computed tomography (CT) venography and CT angiography to exclude a cerebral venous sinus thrombosis and an arteriovenous malformation or fistula, respectively.

**Figure 2:**
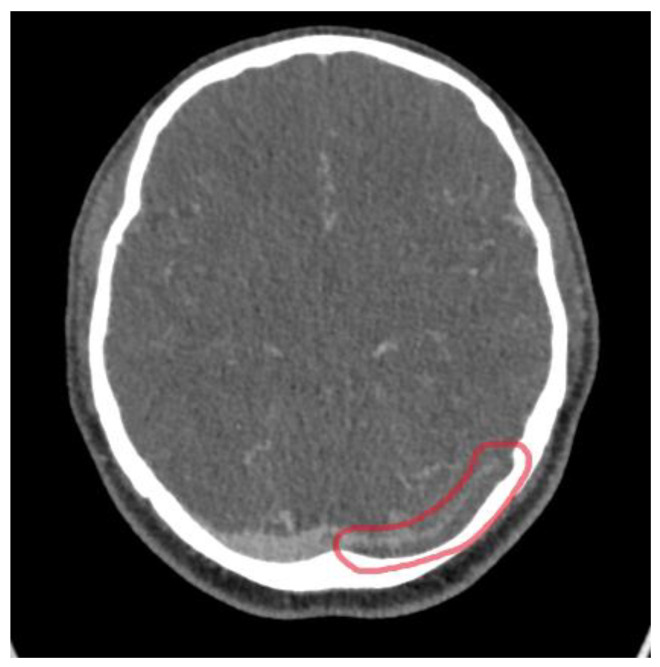
CT venogram intracranial showing a filling defect in the left transverse sinus (red circle).

**Figure 3:**
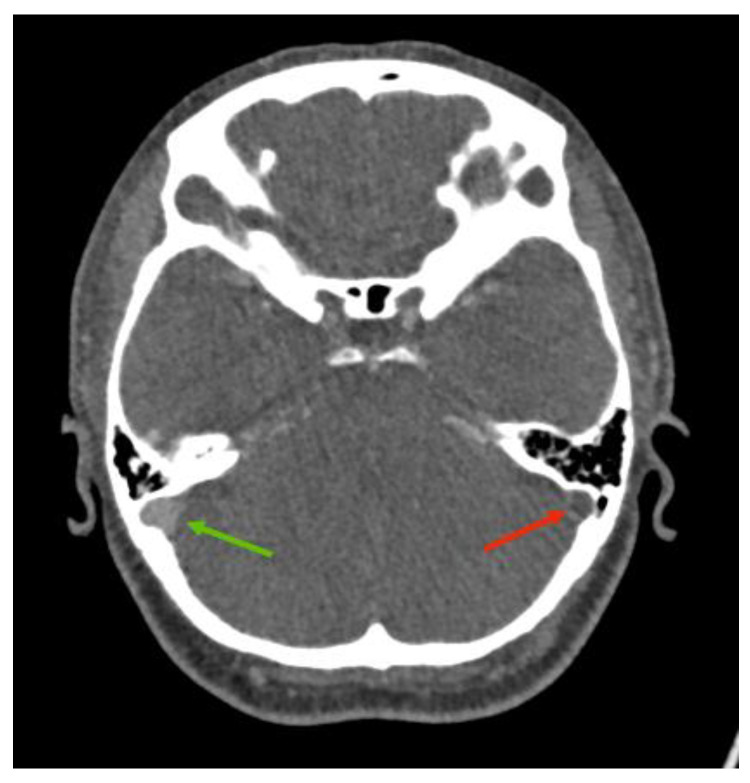
CT venogram intracranial showing a filling defect in the left sigmoid sinus (red arrow) in comparison to the normal right sigmoid sinus filled with contrast media (green arrow).

The CT venogram revealed a filling defect involving the left transverse and sigmoid sinuses extending to the jugular bulb in keeping with cerebral venous sinus thrombosis, as well as cortical venous haemorrhage (see Figure 2 and Figure 3). The CT angiography did not find any intrinsic vascular abnormalities.

The findings were discussed with the nearest neurology team, who recommended initiating the patient on a therapeutic dose of heparin (1.5 mg/kg/day). The patient was subsequently admitted to the neurology unit for approximately two weeks, during which she required two blood transfusions due to a further decline in haemoglobin (Hb) levels below 70 g/L.

**Figure 4:**
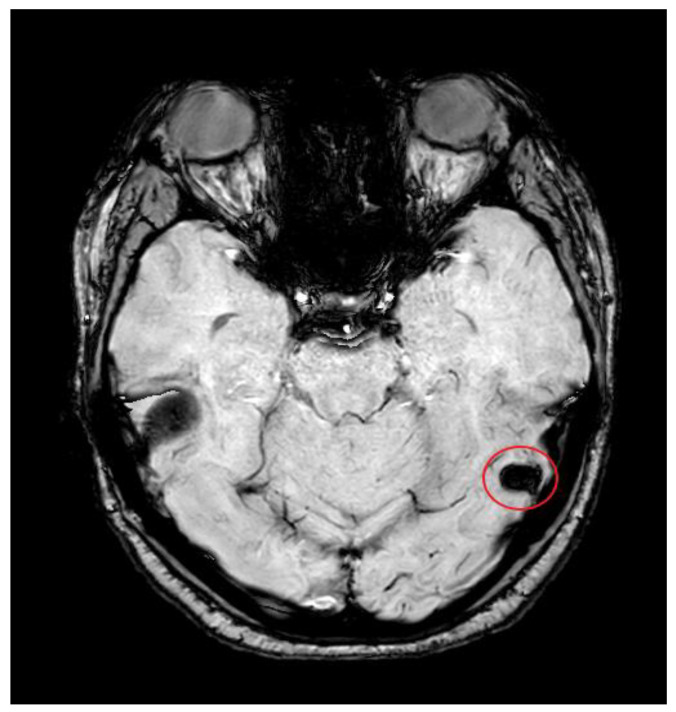
MRI head scan showing a so-called blooming artefact (red circle), indicating thrombosis of the lateral tentorial sinus on the left side with thrombosis extending into the vein of Labbe, resulting from the previously described thrombosis of the left.

Additionally, a magnetic resonance imaging (MRI) head was performed to exclude parenchymal abnormalities or venous infarcts and showed a left transverse sinus, sigmoid sinus, and jugular bulb thrombosis. There was secondary thrombosis of the left lateral tentorial sinus and the vein of Labbe with venous hypertension and vasogenic oedema within the left middle temporal lobe and the fusiform gyrus (see Figure 4).

To address her menorrhagia, the gynaecology team started her on leuprorelin acetate (3.75 mg monthly via subcutaneous injection), a gonadotropin-releasing hormone (GnRH) analogue, to induce a state of menopause.

She was discharged on levetiracetam, which was discontinued after one month. The patient did not experience any further seizures three months after admission. She will continue follow-up appointments in the neurology clinic for another two years.

She was discharged on a therapeutic dose of heparin with advice to continue it for at least six months in total. She has had multiple follow-ups with gynaecology since discharge, including a hysteroscopy, and they are considering her for endometrial ablation due to fibroids. She is clinically stable with no neurological sequelae till the date of writing this report.

## 3. Discussion

### 3.1. Pathophysiology of TXA-Induced CVST

This case highlights a rare but serious complication potentially linked to TXA use, manifesting as cerebral venous sinus thrombosis (CVST) complicated by intracranial haemorrhage (ICH) and new-onset generalised tonic–clonic seizure. While TXA is generally well-tolerated and widely used for the treatment of menorrhagia, clinicians must be aware of its pro-thrombotic potential, especially in patients with additional risk factors.

The pathophysiology of CVST in the context of TXA use is multifactorial. TXA’s anti-fibrinolytic properties may contribute to thrombus formation within the cerebral venous system, particularly in the transverse and sigmoid sinuses, as seen in this patient [13,14]. There is ongoing debate regarding whether the thrombotic effects of TXA are dependent on its synthetic lysine derivative structure. Tenborn et al. [15] suggest that TXA competes for the lysine-binding site on plasminogen, inhibiting its interaction with fibrin and thereby preventing thrombus formation. Nonetheless, large systematic reviews and meta-analyses involving over 100,000 patients have not demonstrated a significant increase in thrombotic events with TXA use, especially when administered at standard therapeutic doses [5,8]. Notably, pharmacovigilance data and isolated case reports have suggested possible thrombotic risks, including rare occurrences of CVST [9,10,11,12]. Therefore, a clear distinction must be made between a possible association and a direct causal relationship. The present case adds to the limited reports suggesting that TXA might contribute to thrombosis under specific circumstances.

It is important to consider other confounding factors in this patient. She had a recent history of norethisterone use, a progesterone-only contraceptive that has been linked to an increased thrombotic risk, although the risk appears to normalise approximately three months after discontinuation [16]. The patient had stopped norethisterone five weeks prior, suggesting a possible residual prothrombotic effect. Moreover, iron deficiency anaemia (IDA) secondary to menorrhagia is recognised as a hypercoagulable state, potentially contributing to thrombus formation by altering blood viscosity and red blood cell deformability [17]. These factors likely created a prothrombotic milieu in which TXA’s antifibrinolytic action may have acted as a precipitant rather than the sole cause of CVST.

In terms of complication, CVST can lead to increased venous pressure, decreased cerebrospinal fluid absorption, and localised venous congestion [18]. This cascade can result in parenchymal injury, manifesting as either venous infarction or intracerebral haemorrhage (ICH), particularly in cortical regions, such as the left temporal lobe in this case [18]. The venous congestion and subsequent disruption of the blood–brain barrier can allow red blood cells to extravasate, resulting in haemorrhagic transformation as observed in this patient [18,19].

### 3.2. Imaging Role

Neuroimaging was pivotal in confirming the diagnosis of CVST in this patient. Initial CT imaging performed within an hour from the onset of the seizure revealed acute haemorrhagic foci in the left temporal lobe, raising suspicion for an intracranial pathology. Subsequent CT venography identified filling defects consistent with thrombosis of the left transverse and sigmoid sinuses extending into the jugular bulb, while CT angiography excluded arteriovenous malformations. MRI further characterised the thrombosis, revealing secondary involvement of the lateral tentorial sinus and vein of Labbe with associated venous hypertension and vasogenic oedema. The multimodal imaging approach was essential in differentiating CVST from other causes of seizure and headache, facilitating targeted management.

### 3.3. Management Implications

Clinically, the presentation with “the worst headache of one’s life” and subsequent seizure is classical for CVST [20]. Headaches are seen in up to 90% of patients with CVST [21], while seizures are seen in up to 40% of CVST cases and may be the first sign of intracranial pathology [22]. The delayed recognition of these symptoms can be detrimental, as the risk of secondary complications such as ICH increases. Notably, this patient’s symptoms escalated during the second course of TXA, which may have exacerbated the thrombotic process.

This patient’s blood showed elevated C-reactive protein (CRP) and leukocyte counts on presentation. These raised inflammatory markers can be nonspecific and often occur in acute neurological events, including seizures and CVST. The systemic inflammatory response may reflect secondary processes related to venous congestion, parenchymal injury, or seizure activity rather than infection [23]. Clinicians should interpret these markers cautiously, correlating them with clinical and imaging findings to avoid misdiagnosis or unnecessary interventions. The management of CVST complicated by ICH requires careful balancing of risks and benefits. Despite concerns about anticoagulation in the presence of intracerebral haemorrhage, therapeutic anticoagulation with heparin remains the standard of care, supported by clinical guidelines and evidence demonstrating reduced morbidity and mortality [18,24,25]. The patient’s prompt initiation of low molecular weight heparin and antiepileptic treatment with levetiracetam contributed to a favourable clinical outcome, with seizure freedom maintained at three months post-admission. The rationale for antiepileptic medication in a patient with known CVST was to reduce the threshold for further seizure while on anticoagulation. No electroencephalography (EEG) was indicated in the follow-up period, as an underlying cause of seizure has been identified according to the NICE guideline [26].

This case highlights the importance of evaluating non-hormonal alternatives for managing menorrhagia in patients with anaemia. The initiation of leuprorelin acetate represents a shift toward a safer long-term strategy, particularly in patients who may be predisposed to hypercoagulable states [27], and for patients planned for endometrial ablation [28].

## 4. Conclusions

This case underscores the rare but potentially life-threatening complication of cerebral venous sinus thrombosis and intracerebral haemorrhage following the use of TXA for menorrhagia. Although TXA is widely regarded as safe, its anti-fibrinolytic properties can precipitate thrombotic events, particularly in patients with underlying or transient prothrombotic risk factors. The presentation of a severe headache and a first-time seizure in this context should prompt immediate neuroimaging to rule out CVST and associated complications. Early recognition and appropriate intervention, including anticoagulation and seizure management, are essential for favourable outcomes. Clinicians should maintain a high index of suspicion and consider safer alternatives for managing heavy menstrual bleeding in patients at risk.

## Figures and Tables

**Table 1:** Symptom chronology.

Day	Clinical Event/Symptom	Details
Day 7	Initiation of TXA	1 g TDS started for menorrhagia; first 4-day course completed without symptom relief.
Day 4	Second course of TXA begun	Another 3-day course prescribed (same dose) due to ongoing bleeding.
Day 3	Headache onset	Described as “worst headache of life”—left-sided, throbbing, 10/10, radiating to neck. Associated with nausea, floaters, blurred vision, and neck stiffness.
Day 0	Seizure and hospital presentation	Generalised tonic–clonic seizure while driving; no neurologic deficit post-ictal. Brought to the emergency unit.

## Data Availability

No new data were created or analyzed in this study. Data sharing is not applicable to this article.
